# Overexpression of microRNA-634 suppresses survival and matrix synthesis of human osteoarthritis chondrocytes by targeting PIK3R1

**DOI:** 10.1038/srep23117

**Published:** 2016-03-14

**Authors:** Xu Cui, Shaojie Wang, Heguo Cai, Yuan Lin, Xinpeng Zheng, Bing Zhang, Chun Xia

**Affiliations:** 1 Zhongshan Hospital, Xiamen University, Xiamen, Fujian, 361000, China; 2 Medical School, Xiamen University, Xiamen, Fujian, 361102, China

## Abstract

Osteoarthritis (OA) is a degenerative disease characterized by deterioration of articular cartilage. Recent studies have demonstrated the importance of some microRNAs in cartilage damage. The aim of this study was to identify and characterize the expression of microRNA-634 (miR-634) in normal and OA chondrocytes, and to determine its role in OA pathogenesis. Human normal and OA chondrocytes obtained from patients were cultured *in vitro*. Transfection with miR-634 mimic or inhibitor was employed to investigate the effect of miR-634 on chondrocyte survival and matrix synthesis, and to identify miR-634 target. The results indicated that miR-634 was expressed at lower level in high grade OA chondrocyte compared with normal chondrocytes. Overexpression of miR-634 could inhibit cell survival and matrix synthesis in high grade OA chondrocytes. Furthermore, miR-634 targeted PIK3R1 gene that encodes the regulatory subunit 1 of class I PI3K (p85α) and exerted its inhibitory effect on the phosphorylation of Akt, mTOR, and S6 signal molecules in high grade OA chondrocytes. Therefore, the data suggested that miR-634 could suppress survival and matrix synthesis of high grade OA chondrocytes through targeting PIK3R1 gene to modulate the PI3K/Akt/S6 and PI3K/Akt/mTOR/S6 axes, with important implication for validating miR-634 as a potential target for OA therapy.

Osteoarhtritis (OA) is a degenerative joint disease characterized by deterioration in the integrity of cartilage and accompanied by pain and disability. Although multiple factors are implicated in OA etiology, chondrocyte, the single cell type present in the mature cartilage, dominates the degenerative process of cartilage^1^. For example, chondrocyte death by apoptosis is associated with the initiation and severity of articular cartilage degradation^2^. Matrix metalloproteinases(MMPs) and aggrecanase secreted by chondrocytes contribute to cartilage degradation^3^,^4^. Several lines of evidence have been proposed to suggest that OA etiology is associated with genetic component and heritability^5^,^6^, and that the alterations of gene expression in chondrocyte are involved in synthesis and degradation of cartilage, including microRNAs (miRNAs)^7^,^8^,^9^,^10^,^11^.

MiRNAs are small (17–25 nucleotide long) non-coding RNA molecules generated from long primary transcripts(primiRNAs) through multiple processing steps including the removal of the loop region of the hairpin by Dicer, the RNase III, which is essential for generation of mature miRNAs^12^. MiRNAs could bind to complementary sequences on target mRNA transcripts and silence their gene expression, resulting in translational repression or target degradation^13^. Approximately one-third of all mammalian genes are regulated by miRNAs^11^. Kobayashi Tatsuya *et al.* have already found that dicer-dependent pathways regulate chondrocyte proliferation and differentiation^14^. Subsequently, differential expression of some miRNAs between normal and OA cartilage has been demonstrated. Jones S.W. *et al.* have identified 17 human miRNA that showed greater than 4-fold differential expression between OA and normal cartilage^15^. Silvia Díaz-Prado1 *et al.* have found that 7 human miRNAs have been identified to express differentially in normal and OA cartilage^16^. These studies implicated the possibility of the involvement of miRNA in OA pathogenesis. Furthermore, some miRNAs were found to promote or suppress chondrocyte events in OA pathogenesis. Overexpression of hsa-miR-148a promotes cartilage production and inhibits cartilage degradation by OA chondrocytes^17^. MicroRNA-33a regulates cholesterol synthesis and cholesterol efflux-related genes in OA chondrocytes^18^. MiR-149 is down-regulated in OA chondrocytes, and this decrease seems to be correlated to increased expression of pro-inflammatory cytokines such as TNFα, IL1β, and IL6^19^. MicroRNA-125b regulates the expression of aggrecanase-1 (ADAMTS-4) in human OA chondrocytes^20^. Therefore, miRNAs could exert positive or negative effect on OA chondrocyte metabolism. The identification of the effect of miRNA on chondrocyte metabolism is beneficial to revealing OA etiology and providing a potential approach for OA therapy.

Recently, miR-634 was demonstrated to be up-regulated in human normal chondrocytes and down-regulated in OA chondrocytes^16^. Using bioinformatics, we found that miR-634 has a seed-matched sequence in 3′-UTR of human PIK3R1, which encodes the regulatory subunit 1 of class I PI3K (p85α)^21^. PI3K is activated by growth factors and hormones, such as epidermal growth factor receptor (EGFR) and insulin growth factor-1(IGF1). Activated class I PI3K converts PtdIns (4, 5) P2 (PIP2) to PIP3 by phosphorylating the hydroxyl group of the inositol ring of the former at the 3-position. The PIP3 then acts as a second messenger to trigger a downstream signaling cascade that is comprised of Akt, mTOR, and other proteins^22^,^23^. Other authors’ and our previous studies have shown that the alternation of those signal molecules is essential for OA development^24^,^25^,^26^,^27^,^28^. However, the role of miR-634 in OA pathogenesis is not revealed.

In this study, the expression of miR-634 and its target gene were investigated in human normal and OA chondrocytes. The effect of miR-634 on survival and matrix metabolism were then detected in OA chondrocytes. Our findings suggested that miR-634 could be a novel regulator of chondrocyte metabolism, including survival and matrix synthesis, by targeting PIK3R1 gene that modulated the PI3K/Akt/S6 and PI3K/Akt/mTOR/S6 axes in OA chondrocytes.

## Results

### The expression of miR-634 in human normal and OA chondrocytes

**Figure 1:**
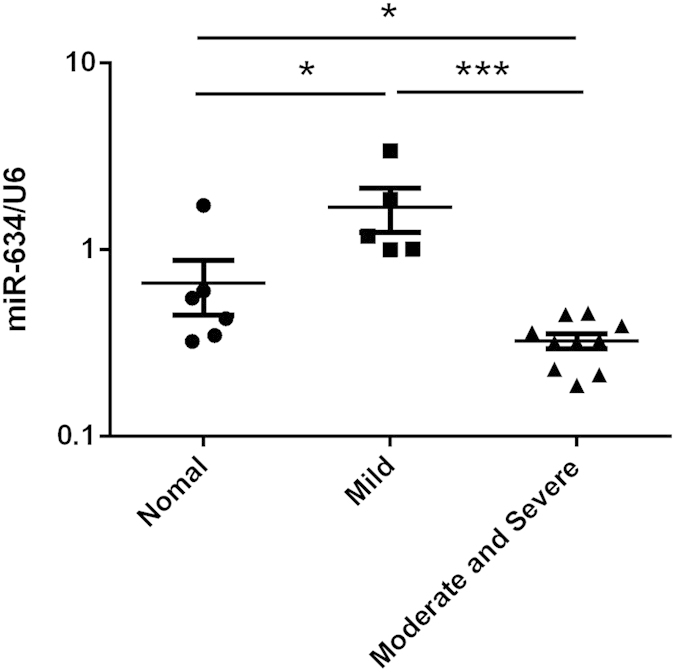
The expression of miR-634 in human normal and OA chondrocytes. Normal chondrocytes obtained from 6 donors with amputation operation and OA chondrocytes obtained from 15 patients who underwent total joint arthroplasty were cultured. All cartilage samples were divided into 3 groups according to a Kellgren/Lawrence Criterion: normal cartilage (K/L, Grade 0) for Normal group, low grade OA cartilage (K/L Grade I & II) for Mild group, and high grade OA cartilage (K/L, Grade III & IV) for Moderate and Severe group. The gene expression of microRNA-634 was measured using qPCR technique as described in the Materials and Methods. The values represent the mean ± SEM of three independent experiments, each yielding similar results (*P < 0.05, ***P < 0.001).

All cartilage samples were divided into 3 groups according to a Kellgren/Lawrence Criterion^29^,^30^: normal cartilage (K/L, Grade 0) for Normal group, low grade OA cartilage (K/L Grade I & II) for Mild group, and high grade OA cartilage (K/L, Grade III & IV) for Moderate and Severe group. Cells were cultured, followed by the detection of the gene level using Quantitative real-time PCR (qPCR) technique. As shown in Fig. 1, the gene level of miR-634 in Moderate and Severe group was the lowest in the three groups, while the gene level of miR-634 in Mild group was the highest in the three groups (Fig. 1, *P < 0.05, ***P < 0.001). The data thus indicated that the level of miR-634 in OA chondrocytes increased at low grade OA chondrocytes, while it decreased at high grade OA chondrocytes, compared with normal chondrocytes.

### The effect of miR-634 on matrix synthesis of human OA chondrocytes

**Figure 2:**
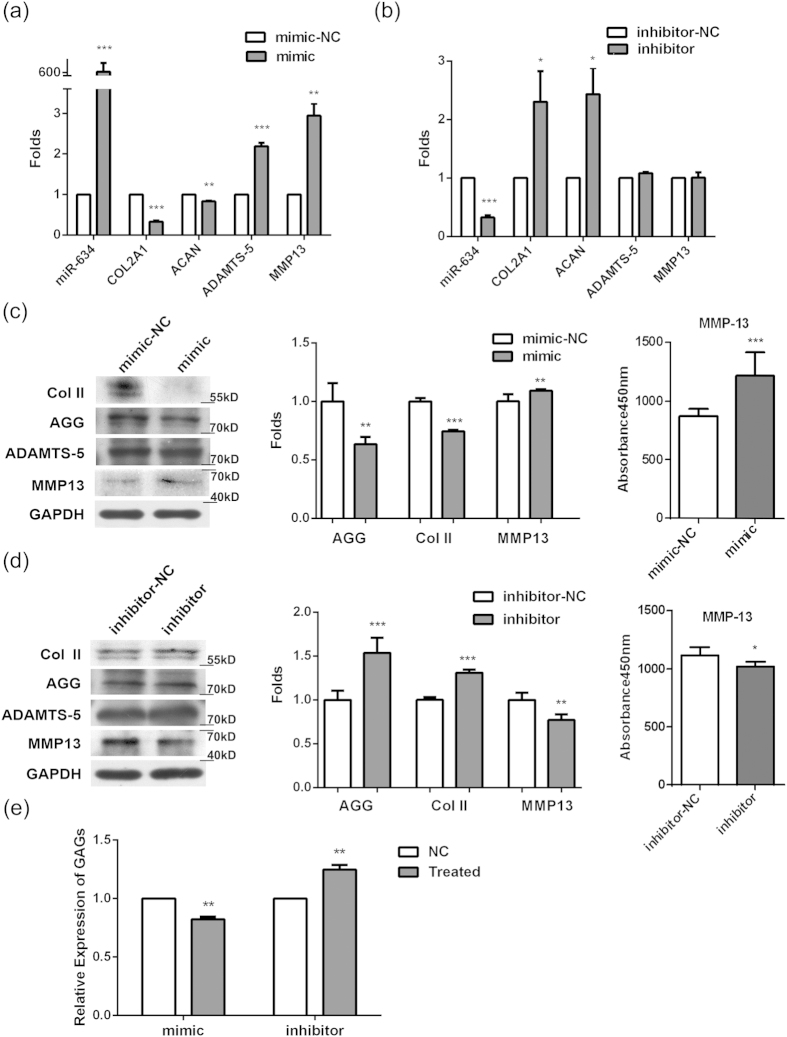
The effect of miR-634 on matrix synthesis of human OA chondrocytes. Cells were transfected with miR-634 mimic or miR-634 inhibitor and their negative control for 48~96 h, respectively. (**a**,**b**) The mRNA levels of COL2A1, ACAN, ADAMTS-5, and MMP13 were measured by qPCR technique as described in the Materials and Methods. (**c**,**d**) The left and middle panels: the protein levels of Col II, AGG, ADAMTS-5, MMP13, and GAPDH were measured by western blotting analysis using anti-Col II (1:2000), AGG (1:1500), ADAMTS-5 (1:2000), MMP13 (1:1500), and GAPDH (1:20000) antibodies under the same experimental conditions (10% SDS-PAGE) as described in the Materials and Methods. The blots were normalized to an endogenous protein (GAPDH). The above blots are cropping blots, and the full-length blots are presented in Supplementary Figures 1–3. The right panel: the expression of MMP13 in extracellular matrix in OA chodnrocytes was detected with ELISA as described in the Materials and Methods. (**e**) The relative level of GAGs was measured by DMMB assay according to the Materials and Methods. The values represent the mean ± SEM of three independent experiments, each yielding similar results (*P < 0.05, **P < 0.01, ***P < 0.001).

Human OA chondrocytes of Moderate and Severe group (high grade OA) cultured were chosen to investigate the role of miR-634 in regulating matrix synthesis of OA chondrocytes, as it was formidable to get the articular cartilage of Normal and Mild groups (low grade OA). After high grade OA chondrocytes were transfected with miR-634 mimic, miR-634 inhibitor, and their negative control (NC) for 48~96 h, respectively, the mRNA/protein levels of matrix synthesis biomarkers, including COL2A1/Col II, ACNA/AGG, ADAMTS-5/ADAMTS-5, and MMP13/MMP13, were detected using Real-time Quantitative PCR (qPCR) technique and western blotting analysis, respectively. The results of qPCR showed that miR-634 mimic dramatically down-regulated the mRNA levels of COL2A1 and ACNA, while the mRNA levels of ADAMTS-5 and MMP13 increased (Fig. 2(a), **P < 0.01, ***P < 0.001, *versus* NC). However, miR-634 inhibitor up-regulated the mRNA levels of COL2A1 and ACNA without the alteration of ADAMTS-5 and MMP13 in mRNA levels (Fig. 2(b), *P < 0.05, ***P < 0.001, *versus* NC). The results of western analysis manifested that the protein levels of AGG and Col II decreased, with the increase in MMP13 protein level in OA chondrocytes transfected with miR-634 mimic (the left & middle panel in Fig. 2(c), **P < 0.01, ***P < 0.001, *versus* NC). MiR-634 inhibitor led to the increase in AGG and Col II protein levels with the decrease of MMP13 protein level (the left panel in Fig. 2(d), **P < 0.01, ***P < 0.001, *versus* NC). Neither miR-634 mimic nor miR-634 inhibitor had effect on ADAMTS-5 protein expression. Moreover, the results of ELISA showed that the variation tendency of MMP13 protein expression in extracellular matrix of OA chondrocytes transfected with mimic or inhibitor of miR-634 (the right panel in Fig. 2(c,d), *P < 0.05, ***P < 0.001) was in accordance with that of intracellular MMP13 protein expression (the left & middle panel in Fig. 2(c,d)). Additionally, the released concentrations of GAGs that is a vital component of extracellular matrix declined in OA chondrocytes transfected with miR-634 mimic, while miR-634 inhibitor up-regulated the released concentrations of GAGs using DMMB assay (Fig. 2(e), **P < 0.01, *versus* NC), similar to the variation tendency of AGG using western blotting analysis (Fig. 2(c,d)). Together, the data indicated that the overexpression of miR-634 could suppress matrix synthesis in high grade OA chondrocytes.

### The effect of miR-634 on survival of human OA chondrocytes

**Figure 3:**
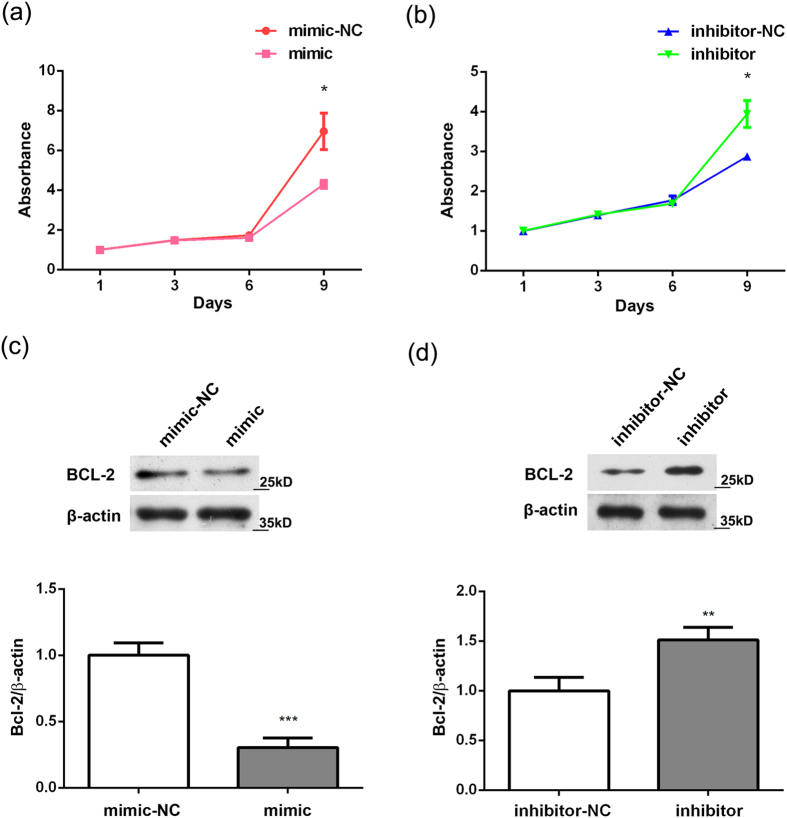
The effect of miR-634 on survival of human OA chondrocytes. Cells were transfected with miR-634 mimic or miR-634 inhibitor and their negative control for 72 h or 96 h. (**a,b**) The proliferation rate of cells was measured by CCK-8 assay as described in the Materials and Methods. (**c**,**d**) The protein levels of Bcl-2 and β-actin were measured by western blotting analysis using Bcl-2(1:2000) and β-actin (1:20000) antibodies under the same experimental conditions (12% SDS-PAGE) as described in the Materials and Methods. The above blots were cropping blots and normalized to an endogenous protein (β-actin). The values represent the mean ± SEM of three independent experiments, each yielding similar results (*P < 0.05, **P < 0.01, ***P < 0.001).

As chondrocyte apoptosis is one of crucial elements in OA pathogenesis, the effect of miR-634 on chondrocyte survival was investigated using CCK8 assay and western blotting analysis, respectively. The results of CCK8 assay showed that miR-634 mimic inhibited cell proliferation of OA chondrocyte at the 9^th^ day (Fig. 3(a), *P < 0.05, *versus* NC). In contrast, miR-634 inhibitor promoted cell proliferation of OA chondrocyte at the same time (Fig. 3(b), *P < 0.05, *versus* NC). Meanwhile, the protein level of Bcl-2 decreased in OA chondrocytes transfected with miR-634 mimic (Fig. 3(c), ***P < 0.001, *versus* NC) and vice versa in OA chondrocytes transfected with miR-634 inhibitor (and 3(d), **P < 0.01, *versus* NC). Overall, the overexpression of miR-634 could inhibit survival of high grade OA chondrocytes.

### MiR-634 targets PIK3R1 in human OA chondrocytes

**Figure 4:**
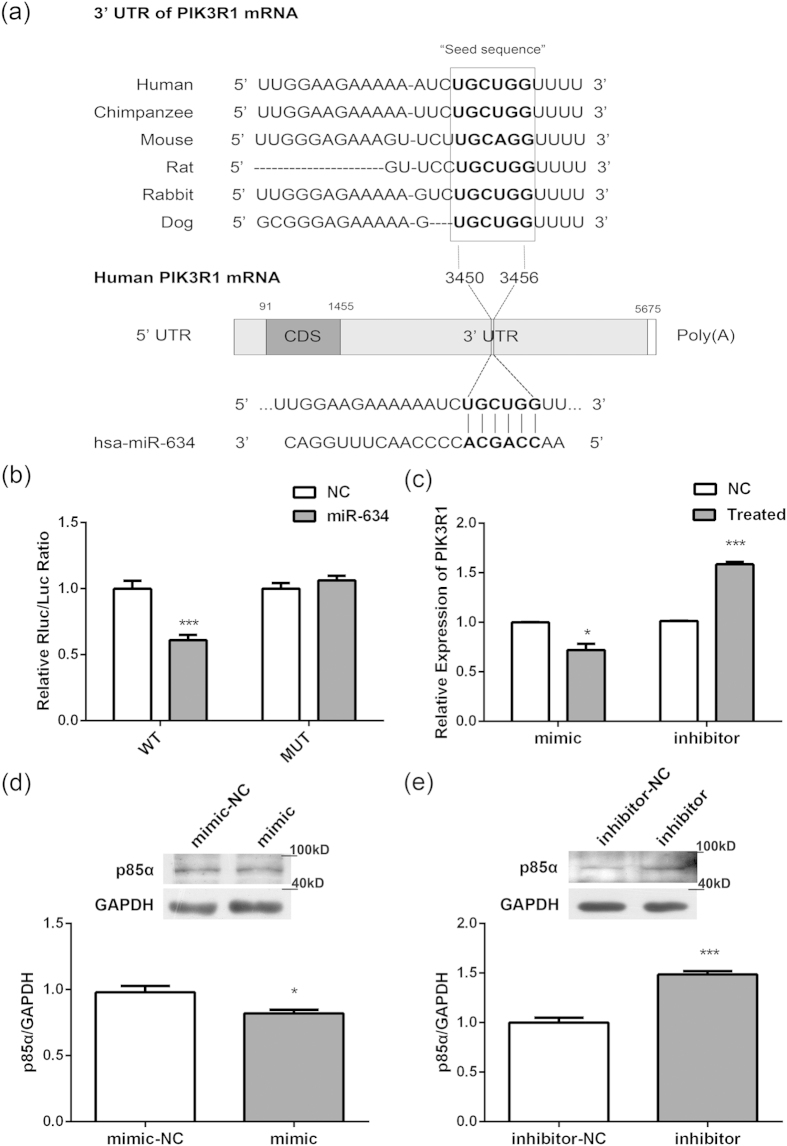
MiR-634 targets PIK3R1 in human OA chondrocytes. (**a**) The schematic shows that the 3′UTR of PIK3R1 contains the miR-634 seed sites. (**b**) HEK293 cells were transiently co-transfected with the pmiR-PIK3R1-WT (wild-type 3′UTR) or pmiR-PIK3R1-MUT (mutant sites) of PIK3R1 REPORT™ reporter plasmids, and miR-634 mimic, respectively, All cells were harvested at 48 h after transfection, and then Luciferase activity were measured by dual-luciferase reporter assay as described in the Materials and Methods. (**c–e**) Cells were transfected with miR-634 mimic or miR-634 inhibitor and their negative control for 72 h or 96 h. (**c**) The mRNA levels of PIK3R1 in OA chondrocytes were measured using qPCR technique as described in the Materials and Methods. (**d,e**) The protein expression of p85α in OA chondrocytes were detected by western blotting analysis using p85α (1:2000) and GAPDH (1:20000) antibodies under the same experimental conditions (10% SDS-PAGE) as described in the Materials and Methods. The blots were cropping blots and normalized to an endogenous protein (GAPDH). The values represent the mean ± SEM of three independent experiments, each yielding similar results (*P < 0.05, ***P < 0.001).

Diana-MICROT, miRDB, TargetMiner, and TargetScan, were chosen to determine the possible target genes of miR-634. It was found that 3′UTR of PIK3R1, which encodes class I PI3K regulatory subunit 1 (α) (PIK3R1, alias: p85-α)^21^, contained the miR-634 seed sites and that sequences were highly conserved among diverse species (Fig. 4(a)). To determine whether miR-634 could target PIK3R1, HEK293 cells were transiently co-transfected with the pmiR-PIK3R1-WT (wild-type 3′UTR) or pmiR-PIK3R1-MUT (mutant sites) of PIK3R1 REPORT™ reporter plasmids, and miR-634 mimic, respectively, prior to dual-luciferase analysis. The results showed that miR-634 could down-regulate the luciferase activity of pmiR-PIK3R1-WT plasmid, but not that of pmiR-PIK3R1-MUT, manifesting that PIK3R1 was one of miR-634 targets (Fig. 4(b), ***P < 0.001, *versus* NC). Furthermore, the mRNA level of PIK3R1 was detected using qPCR technique. The results of Fig. 4(c) displayed that miR-634 mimic down-regulated the mRNA level of p85α, while miR-634 inhibitor up-regulated the mRNA level of p85α (Fig. 4(c), *P < 0.05, ***P < 0.001, *versus* NC). Similarly, miR-634 mimic down-regulated the protein level of p85α (Fig. 4(d), *P < 0.05, *versus* NC) and vice versa in OA chondrocytes transfected with miR-634 inhibitor (Fig. 4(e), ***P < 0.001, *versus* NC). Consequently, miR-634 could regulate the mRNA and protein level of p85α, indicating that PIK3R1 could be one of target genes inhibited by miR-634 in high grade OA chondrocytes.

### The effect of miR-634 on the PI3K/Akt pathway in human OA chondrocytes

**Figure 5:**
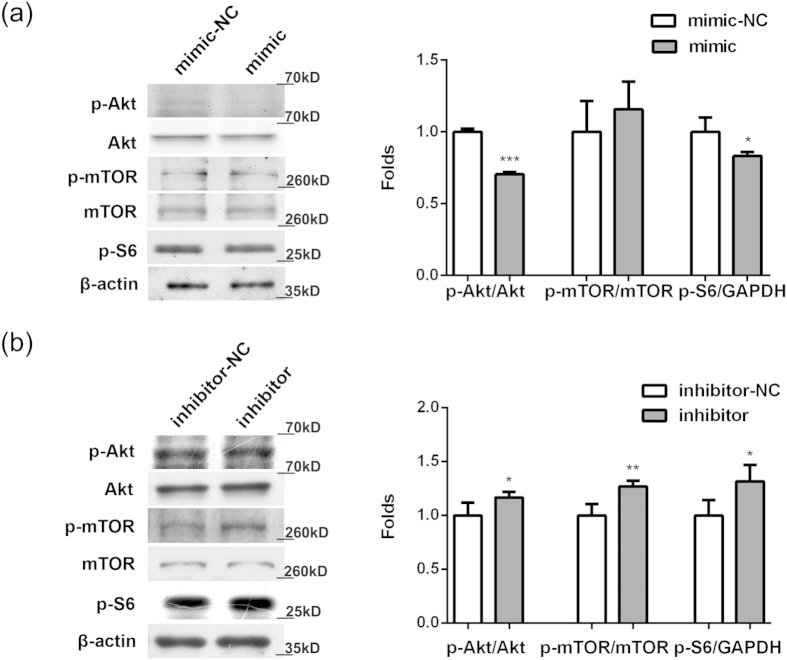
Modulation of miR-634 on the PI3K/Akt pathway. Cells were transfected with miR-634 mimic, miR-634 inhibitor or their negative control for 72 h or 96 h, and then the levels of Akt, p-Akt, mTOR, p-mTOR, p-S6, and GAPDH were measured by western blotting analysis using Akt(1:2000), p-Akt(1:1500), mTOR(1:2000), p-mTOR (1:2000), p-S6(1:2000), and GAPDH(1:20000) antibodies under the same experimental conditions (8% or 10% SDS-PAGE) as described in the Materials and Methods. The blots were cropping blots and normalized to an endogenous protein (GAPDH). (**a**) Cells were transfected with miR-634 mimic. (**b**) Cells were transfected with miR-634 inhibitor. The values represent the mean ± SEM of three independent experiments, each yielding similar results (*P < 0.05, **P < 0.01, ***P < 0.001).

Akt is well known to regulate important cellular events, including cell survival, proliferation, and development, and activated by PI3K. To investigate whether miR-634 has an inhibitory effect on the PI3K/Akt pathway, cells were transfected with miR-634 mimic, miR-634 inhibitor or their negative control in OA chondrocytes, respectively. As showed in Fig. 5(a), miR-634 reduced the levels of p-Akt and p-S6 without the alteration of Akt, mTOR and p-mTOR levels (^*^P < 0.05, ***P < 0.001, *versus* NC). Meanwhile, the level of p-Akt, p-mTOR, and p-S6 increased in OA chondrocytes transfected with miR-634 inhibitor, while total Akt and mTOR did not change (Fig. 5(b), *P < 0.05, **P < 0.01, *versus* NC). These results suggested that the overexpression of miR-634 has an inhibitory effect on the PI3K/Akt pathway, partly associated with mTOR and S6 signal molecules.

## Discussion

MiRNAs have been reported to be expressed differentially in normal and OA chondrocytes^15^,^16^. Amongst the 7 miRNAs (miR-483-5p, miR-149, miR-582-3p, miR-1227, miR-634, miR-576-5p, and miR-641) detected by Silvia Díaz-Prado1 *et al.*, 6 miRNAs were down-regulated in OA chondrocytes, and miR-634 is one of the six miRNAs^16^. Consistent with the above study, our data indicated the down-regulation of miR-634 in high grade OA chondrocyte compared with normal chondrocytes. Therefore, it is confirmed that miR-634 is expressed at the lower level in high grade OA chondrocytes.

Recent studies have demonstrated the involvement of some miRNAs in regulating OA pathological progression. As an example, the decrease of miR-149 in OA chondrocytes seems to be correlated to increased expression of pro-inflammatory cytokines such as TNFa, IL1β and IL6^19^. Especially, the association between miRNA and extracellular matrix of chondrocyte has also been investigated: that microRNA-145 contributes to impaired extracellular matrix in OA cartilage^31^, that microRNA-125b regulates the expression of ADAMTS-4 in human OA chondrocytes^20^, and that microRNA-127-5p is an important regulator of MMP13 in human chondrocytes and may contribute to the development of OA^32^. Here, our data also indicated that miR-634 could regulate the expression of main biological markers in matrix metabolism, such as Col II, AGG, MMP13, and ADAMTS-5 in high grade OA chondrocytes. Meanwhile, miR-634 was involved in regulating chondrocyte survival, similar to the effect of other miRNAs on chondrocyte survival. As an example, miR-146a may lead to an increase of apoptosis rate in chondrocyte^33^. The overexpression of miR-223 stimulates apoptotic cell death in human articular chondrocytes and induces severe cartilage destruction in db/db mice^34^. Hence, we suggested that miR-634 could be involved in regulating survival and matrix synthesis of OA chondrocytes.

However, our data indicated that decreased miR-634 exerted the inhibitory effect on survival and matrix synthesis in high grade OA chondrocytes, seeming to be contrary to conventional deduction that only the higher expressed elements could have an effect on cell events. Based on our data of the high expression of miR-634 in low grade OA chondrocytes, we speculate that the increase of miR-634 may contribute to the development of OA pathogenesis. Whereas, the decrease of miR-634 in high grade OA chondrocytes may be attributed to a spontaneous resistance of chondrocytes to OA pathological microenvironment, or more complicate regulatory mechanism on miR-634. Moreover, the inhibitory effect of the overexpression of miR-634 on cell survival in cancer cells has already been demonstrated: that the overexpression of miR-634 activates the mitochondrial apoptotic pathway by direct concurrent targeting of genes associated with mitochondrial homeostasis, anti-apoptosis, antioxidant ability and autophagy in a model of esophageal squamous cell carcinoma^35^, and that miR-634 is one of the most effective miRNAs to induce apoptosis and inhibit the level of p-Akt in HER2 positive breast cancer^36^. These findings could support our data that miR-634 suppressed survival of OA chondrocytes. The role of miR-634 in the whole OA progression needs to be further studied.

Additionally, some possible target genes of miRNAs involved in OA pathogenesis have been detected out successively. As an example, miR-146a may contribute to OA pathogenesis by increasing VEGF levels and by impairing the TGF-β signaling pathway through targeted inhibition of Smad4 in cartilage^33^. MiR-21 controls the development of OA by targeting GDF-5 in chondrocytes^37^. MiR-210 inhibits an inflammatory signaling pathway, NF-κB, by targeting DR6 in OA^38^. Based on bioinformatics prediction, our results of dual-luciferase and qPCR analysis gave evidence that miR-634 could bind to the complementary sequences in the 3′UTR of PIK3R1 gene, and inhibit PIK3R1mRNA expression in OA chondrocytes. Therefore, we suggested that PIK3R1 is one of the putative target genes inhibited by miR-634.

Amongst the signaling cascades triggered by PIK3R1, the PI3K/Akt pathway is known as one of the most prominent signal cascades to regulate cell survival, differentiation, and development. Other author’s and our previous studies have demonstrated that the PI3K/Akt pathway is involved in OA pathological pathogenesis^24^,^25^,^26^,^39^. As an example, increased expression of the Akt inhibitors TRB3 in OA chondrocytes inhibits insulin-like growth factor 1-mediated cell survival and proteoglycan synthesis^26^. Increased apoptosis in human knee OA cartilage related to the expression of Akt and PKCα^39^. Moreover, activated PI3K/Akt pathway by some extracellular factors could ameliorate cartilage damage. Ginsenoside Rg1 protects chondrocyte from IL-1β-induced mitochondria-activated apoptosis through the PI3K/Akt pathway^40^. The promotion of Akt activity by morroniside could be beneficial to chondrocyte survival^28^. Therefore, the PI3K/Akt pathway could promote chondrocyte survival and matrix synthesis in OA pathogenesis. Here, our findings that the overexpression of miR-634 suppressed the phosphorylation of Akt indicated that miR-634 exerted an inhibitory effect on the PI3K/Akt pathway by targeting PIK3R1 gene. Furthermore, previous studies have emphasized that PI3K/Akt-mediated phosphorylation of S6 could be accomplished in an mTOR-independent or dependent manner, such as Akt/S6 and Akt/mTOR/S6 axes, regulating protein synthesis^41^,^42^,^43^,^44^,^45^,^46^,^47^,^48^. Consistent with these studies, our findings of the effect of miR-634 on the phosphorylation of mTOR and S6 is indicative of the involvement of the PI3K/Akt/S6 (miR-634 mimic) and PI3K/Akt/mTOR/S6 (miR-634 inhibitor) axes in the regulatory mechanism of miR-634. Therefore, miR-634 could target PIK3R1 to inhibit the PI3K/Akt/S6 and PI3K/Akt/mTOR/S6 axes, contributing to the regulatory mechanism of miR-634 on survival and matrix metabolism in high grade OA chondrocytes.

In conclusion, we demonstrated that miR-634 was expressed differentially in the whole OA pathological progression, including higher expression in low grade OA chondrocytes and lower expression in high grade OA chondrocytes, compared with normal chondrocytes. Overexpression of miR-634 could suppress cell survival and matrix synthesis in high grade OA chondrocytes. Furthermore, miR-634 targeted PIK3R1 gene that encodes the regulatory subunit 1 of class I PI3K (p85α) followed by the regulation of the PI3K/Akt/S6 and PI3K/Akt/mTOR/S6 axes in high grade OA chondrocytes. Therefore, the overexpression of miR-634 could inhibit cell survival and matrix synthesis by targeting PIK3R1 to modulate PI3K/Akt signal cascade in high grade OA chondrocytes.

## Materials and Methods

### Reagents

The antibodies against Akt, p-Akt(Ser473), Bcl-2, mTOR, p-mTOR(Ser2448), and p-S6 (Ser235/236) were purchased form Cell Signaling Technology (Beverly, MA,USA). The antibodies against p85α, Col II, ADAMTS-5, and Aggrecan(AGG) were obtained from Sigma-Aldrich(St. Louis, MO, USA) and Abcam(Cambridge, UK), respectively. The antibodies against GAPDH and β-actin were purchased from Santa Cruz Biotechnology (Santa Cruz, CA, USA). Collagenase II and DMEM/F12 were obtained from Gibco (Carlsbad, CA, USA). Unless otherwise specified, the rest of the reagents were purchased from Sigma-Aldrich (St. Louis, MO, USA).

### Patients

Human OA cartilage was obtained from 15 patients (average age 64.40 ± 7.13 years) with OA who underwent total knee replacement surgery. The OA patients were diagnosed according to the American College of Rheumatology criteria, and had not taken non-steroidal anti-inflammatory drugs or steroids for at least 2 weeks prior to surgery or had not any intra-articular injection for at least 1 month prior to surgery. Normal cartilage samples were obtained from 6 patients (average age 42.67 ± 15.41 years) with femoral neck fracture with no known history of OA or RA who underwent total hip replacement surgery according to the previous study^49^. In order to avoid the selection bias, these patients were selected in random. The 21 patients were stratified according to the Kellgren/Lawrence (K.L.) Image Criterion^29^,^30^, and the clinical characteristics were shown in Table 1. The study was approved by our institutional ethics committee (Zhongshan Hospital, Xiamen University (ID no. 20100426, China) according to the principles expressed in the Declaration of Helsinki. All samples were processed after receiving all patient consent and methods were carried out in accordance with the approved guidelines and regulations.

### Human chondrocyte isolation and culture

As described previously^28^,^49^, human articular cartilage was minced to digestion with 0.2% collagenase II in Dulbecco’s modified Eagle’s medium (DMEM). Chondrocytes were maintained in DMEM containing 10% fetal bovine serum (FBS, Gibco, USA) for 24 h at 37 °C. The cells were filtered through a 0.075 mm cell strainer and washed before culturing or miRNA/mRNA isolation with sterile phosphate buffered saline (PBS). First passage chondrocytes were obtained after 2 weeks. All the experiments were done within 3 days of passage 1 culture. During the culture period, cells were incubated at 37 °C in a humidified atmosphere of 5% CO_2_ and 95% air, and the medium was changed every 2 days.

### Transfection

Human chondrocytes were transfected with miR-634 mimic or miR-634 inhibitor (Ribobio, Guangzhou, China) at 30 nM or 200 nM, respectively, using Lipofectamine 3000 according to the manufacturer’s protocol (Invitrogen, USA). MiRNA mimic control or inhibitor control (Ribobio, Guangzhou, China) was used as controls. After transfection for 48–96 h, the cells were used for the following experiments.

### Real-time Quantitative PCR (qPCR)

Total RNAs from cultured chondrocytes were isolated with Trizol reagent (Invitrogen, CA, USA). MiRNA was extracted from cultured chondrocytes using RNAiso for small RNA reagent (TAKARA, JAPAN). RNA samples were reverse-transcribed into cDNA with the PrimeScript RT Master Mix (total RNA) and the SYBR PrimeScript miRNA RT-PCR Kit (TAKARA, JAPAN). Real-time PCR was performed with gene-specific primers (Table 2), the SYBR Premix Ex Taq II, and the SYBR PrimeScript miRNA RT-PCR Kit (TAKARA, JAPAN), using the ABI StepOnePlus Sequence Detection System v2.1 (Applied Biosystems, Singapore). The expression of PIK3R1, COL2A1, ACAN, ADAMTS-5, and MMP13 mRNA was quantified with GAPDH mRNA expression as endogenous control. The expression of miRNA was quantified with RNU6B control. Quantification of the relative levels was determined by the ΔΔCt method according to previous studies^19^,^50^.

### MiRNA target prediction and luciferase reporter assay

Diana-MICROT (http://www.microrna.gr/microT-CDS), miRDB (http://mirdb.org), TargetMiner (http://www.isical.ac.in) and TargetScan (http://www.targetscan.org) were used to predict the miR-634 targets. The miR-634 target region of the PIK3R1-3′UTR was inserted into the pmiR-RB-Report vector and named pmiR-PIK3R1-WT (RiboBio, Guangzhou, China). As a control, the plasmid containing the mutant sequence of the miR-634 target region of the PIK3R1 3′UTR was also inserted into the pmiR-RB-Report vector and named pmiR-PIK3R1-MUT. HEK293 cells were transiently co-transfected with the 100 ng pmiR-PIK3R1-WT or pmiR-PIK3R1-MUT and 30 nM miR-634 mimic or its negative control using Lipofectamine 3000 (Invitrogen, CA, USA) in 24-well plates, respectively. Each experiment was repeated 3 times. 48 hours after transfection, luciferase activity was determined with Dual-Luciferase® Assay System according to manufacturer’s protocol (Promega, WI, USA). Luminescence was measured using Modulus™ II (Turner Biosystems, USA).

### Western blotting analysis

Cells collected by centrifugation were lysed as previously described^51^. Protein extracts were subjected to SDS-PAGE (8–12%, according to the molecular weight of protein) and transferred to PVDF membranes (Millipore, MA, USA) as described in previous studies^51^,^52^. After blocking with 5% nonfat milk in TBST (Tris-buffered saline plus 0.1% Tween 20), the membranes were incubated with primary antibodies against p85α, Col II, AGG, mTOR, p-mTOR, Akt, p-Akt, Bcl-2, p-S6, β-actin, and GAPDH. After washing, the membranes were incubated with secondary anti-mouse or anti-rabbit antibodies, respectively. The signal was monitored using a chemiluminescent detection system according to the manufacturer’s protocol (Millipore, MA, USA).

### Determination of MMP13 by ELISA

The cell culture supernatant was collected and added to each ELISA plate well pre-coated with anti-human MMP13 polyclonal antibody. The level of MMP13 in the culture supernatant was then measured by the human MMP13 kit (Neobioscience, Shenzhen, China) according to the manufacturer’s instruction and previous study^53^.

### Dimethylmethylene blue assay

The glycosaminoglycan content of the chondrocytes in culture was determined by the dimethylmethylene blue (DMMB) method as described in previous study^54^. In brief, the samples were digested with 2 mg/ml papain in a buffer of HEPES buffered saline solution (HBS), for 12 h at 60 °C. Shark chondroitin sulfate purchased from Sigma-Ardrich (St. Louis, MO, USA) was used as a standard (0–70 ng). On a 96-well plate, 20 μl of blank papain solution, each standard and sample dilution (triplicate) were added. 200 μl of DMMB solution was added to each well, before reading the plate at 530 nm (spectrophotometer) within 10 minutes. The amount of GAGs was normalized by RNA content measured by infinite M200 (Tecan, Swit).

### Cell proliferation analysis

Cells after transfection for 48 h were plated in 96-well plates (1 × 10^3^ cells/well) and cultured for different time (1, 3, 6, 9 d), respectively. 10 μl Cell Counting Kit-8 (CCK8) solution (Beyotime, Shanghai, China) was then added per well and the cultures were incubated at 37 °C for a further 3 h as described in previous study^55^. The end product was quantified spectrophotometrically at a wavelength of 450 nm. The OD values correspond to the number of viable cells^28^,^54^.

### Statistical analysis

Experimental data were formulated as the means ± S.E.M. of triplicate independent samples. The differences between the groups were examined for statistical significance using the unpaired t-test with GraphPad Prism 5 software. A value of p < 0.05 was considered as being significant.

## Additional Information

**How to cite this article**: Xu, C. *et al.* Overexpression of microRNA-634 suppresses survival and matrix synthesis of human osteoarthritis chondrocytes by targeting PIK3R1. *Sci. Rep.* **6**, 23117; doi: 10.1038/srep23117 (2016).

## Supplementary Material

Supplementary Information

## Figures and Tables

**Table 1:** Information of patients with total knee or hip replacement surgery.

Group	Case	Age(mean ± SD)	Sex		K.L. Image Criterion* 29,30					
			M	F	0	I	II	III	IV	V
Normal	6	42.67 ± 15.41	3	3	6					
Mild	5	60.80 ± 9.52	3	2		4	1			
Moderate and Severe	10	66.20 ± 5.29	4	6				7	2	1

^*^K/L = Kellgren/Lawrence; M = male; F = female.

**Table 2:** Primer sequences.

Primer	Sequences
PIK3R1	Forward: GGTGGACGGCGAAGTAAAG
	Reverse: TGAGGGAGTCGTTGTGCTG
COL2A1	Forward: AGCAGGCGTAGGAAGGTCAT
	Reverse: AGAACTAATGGAGCAGCAAGA
ACAN	Forward: CAACTACCCGGCCATCC
	Reverse: GATGGCTCTGTAATGGAACAC
ADAMTS-5	Forward: TGGACCTACCACGAAAGCA
	Reverse: CACAGGCGAGCACAGACAT
MMP13	Forward: CCCCAGGCATCACCATTCAA
	Reverse: CAGGTAGCGCTCTGCAAACT
GAPDH	Forward: GGAAGGTGAAGGTCGGAGTCA
	Reverse: GTCATTGATGGCAACAATATCCACT
