# Supplementary material for: Overexpression of microRNA-634 suppresses survival and matrix synthesis of human osteoarthritis chondrocytes by targeting PIK3R1
Source: Sci Rep. 2016 Mar 14;6:23117. doi: 10.1038/srep23117 (PMC4789801; doi:10.1038/srep23117)
Supplement: Supplementary Information [file srep23117-s1.pdf]

Supplementary data

Overexpression of microRNA-634 suppresses survival and matrix synthesis of human osteoarthritis chondrocytes by targeting PIK3R1

Cui Xu[1,#] Shaojie Wang[1,#] Heguo Cai[1] Yuan Lin[1] Xinpeng Zheng[1] Bing Zhang[2,*] Chun Xia[1,*]

[1] Zhongshan Hospital, Xiamen University, Xiamen, Fujian, 361000, China

[2]Medical School, Xiamen University, Xiamen, Fujian, 361102, China

[#] These authors contributed equally to this work and should be considered as co-first authors.

E-mails: Cui Xu (cuixu666@qq.com); Shaojie Wang (459871798@qq.com); Heguo Cai (442600841@qq.com); Yuan Lin(13860496252@163.com); Xinpeng Zheng(113794258@qq.com).

*Correspondence and requests for materials should be addressed to Bing Zhang (cristal66@xmu.edu.cn) or Chun Xia (chunxia@xmu.edu.cn.).

Supplementary Figure 1

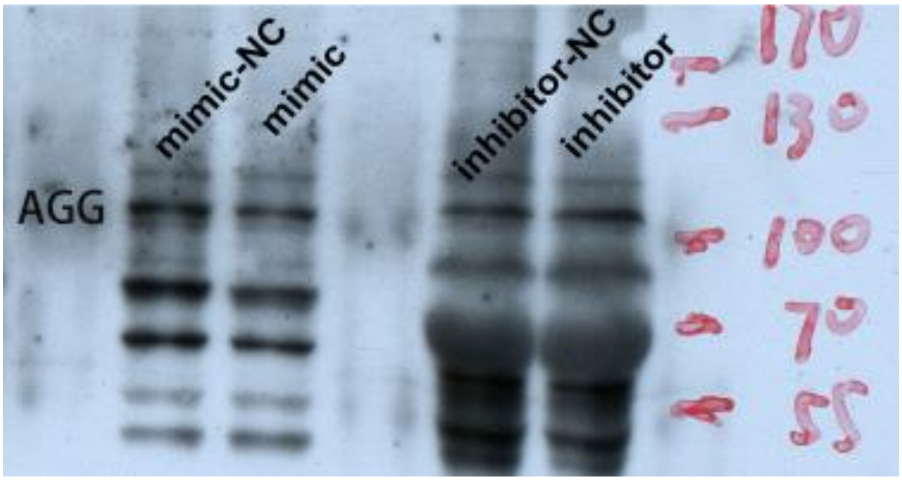

Supplementary Figure 1 The full-length gel indicates the effect of miR-634 on Aggrecan(AGG) expression of human OA chondrocytes.

Supplementary Figure 2

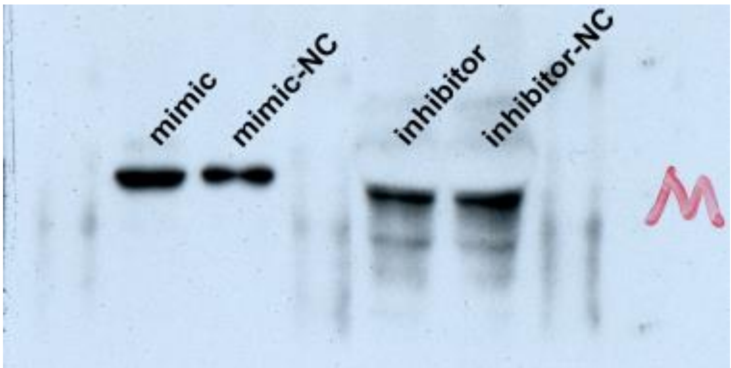

Supplementary Figure 2 The full-length gel indicates the effect of miR-634 on MMP13(M) expression of human OA chondrocytes.

Supplementary Figure 3

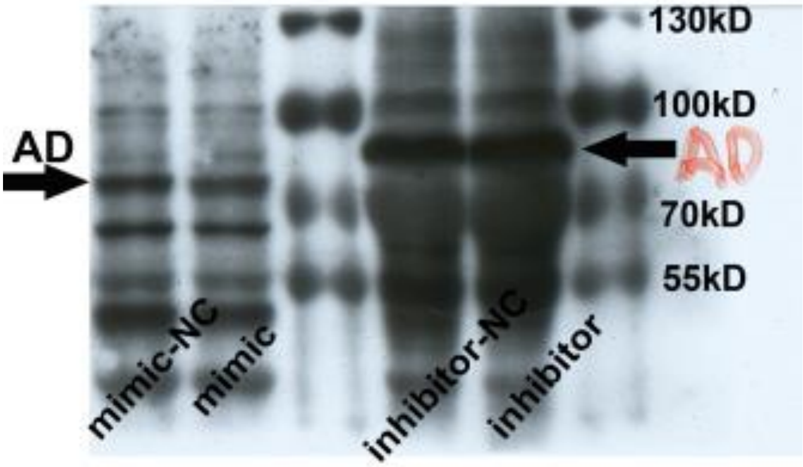

Supplementary Figure 3 The full-length gel indicates the effect of miR-634 on ADAMTS-5(AD) expression of human OA chondrocytes.
